# Tailoring whey protein isolate properties through controlled cold plasma processing: excitation frequency, voltage and time as key variables

**DOI:** 10.1002/jsfa.70245

**Published:** 2025-10-11

**Authors:** Gabriel Oliveira Horta, Paula Zambe Azevedo, Breno Rodrigues de Souza, Sueli Rodrigues, Fabiano André Narciso Fernandes, Daiana Wischral, Paulo Cesar Stringheta, Evandro Martins, Pedro Henrique Campelo

**Affiliations:** ^1^ Department of Food Technology Federal University of Viçosa Viçosa Brazil; ^2^ Department of Food Engineering Federal University of Ceará Fortaleza Brazil; ^3^ Department of Chemical Engineering Federal University of Ceará Fortaleza Brazil

**Keywords:** emerging technologies, processing parameters, excitation frequency, cold plasma, whey protein isolate

## Abstract

**BACKGROUND:**

Cold plasma (CP) is a promising nonthermal technology for tailoring protein structure and functionality. Its ability to induce conformational rearrangements makes it a potential tool for improving the techno‐functional performance of whey protein isolate (WPI). WPI was subjected to CP treatment under varying frequencies (50, 500 and 950 Hz), voltages (10, 15 and 20 kV) and times (10, 15 and 20 min). Analyses included secondary structure (Fourier transform infrared), zeta potential, surface hydrophobicity, carbonyl and free sulfhydryl contents, solubility and curcumin–protein fluorescence binding parameters.

**RESULTS:**

CP increased *α*‐helix and *β*‐sheet contents, promoting protein aggregation. Zeta potential was positively affected by higher frequency but reduced by higher voltage. Surface hydrophobicity consistently increased, while free sulfhydryl content decreased with frequency but increased with time and voltage. Carbonyl content rose under stronger treatments. Solubility decreased across all conditions, attributed to aggregation. Binding affinity (*K*_a_) and number of binding sites (*n*) declined, indicating structural rearrangements and partial denaturation.

**CONCLUSIONS:**

Controlled CP treatment significantly modified WPI structure and functional behavior, reducing solubility and binding affinity while enhancing aggregation. These findings demonstrate the potential of CP as a versatile tool to tailor protein properties for food applications. © 2025 The Author(s). *Journal of the Science of Food and Agriculture* published by John Wiley & Sons Ltd on behalf of Society of Chemical Industry.

## INTRODUCTION

The demand for functional food proteins has surged in recent years, driven by the growing need for ingredients with improved technological properties, such as emulsification, foam formation and gelation. Whey protein isolates (WPIs) stand out in this context due to their interfacial properties, which are crucial for stabilizing food emulsions and foams. However, the natural interfacial characteristics of WPI can be limited in certain food systems, sparking interest in modification techniques that enhance these functionalities.^1^, ^2^, ^3^

Thermal treatments, high hydrostatic pressure, functional group modification or enzymatic hydrolysis are examples of strategies commonly applied for protein modification. However, less aggressive and more efficient alternatives have been attracting industrial interest, demanding strategic advancements. Among the emerging technologies for protein modification, the use of cold plasma (CP) has stood out as a promising approach. This nonthermal processing technology generates a state of ionized matter, composed of electrons, ions, free radicals and other reactive species. When applied to proteins, CP can induce structural modifications, altering their interfacial properties without compromising their functional integrity or nutritional value.^4^

Excitation frequency and plasma voltage are key parameters influencing the efficiency and type of modifications induced in foods. Previous studies concerning aria starch and dry fruits, using different plasma excitation frequencies and voltages, have mainly evaluated their effects on the technological properties of starches, bioavailability of bioactive compounds and antioxidant content.^5^, ^6^ In both cases, the application of a specific excitation frequency contributed to improvements in the parameters studied. Variations in these parameters when applied to food proteins, for example oat and peanut proteins, can affect everything from partial denaturation to protein aggregation, resulting in changes in their adsorption and interface stabilization capacities.^7^, ^8^, ^9^, ^10^ Wang *et al*.^11^ observed that plasma treatment significantly improved the solubility and surface hydrophobicity of proteins extracted from sunflower seeds.

The study presented here aimed to evaluate the effects of CP excitation frequency and voltage on the interfacial properties of WPI at different time intervals. Each process variable was evaluated at three levels: excitation frequency (50, 500 and 950 Hz), voltage (10, 15 and 20 kV) and time (10, 15 and 20 min). Finally, the interaction between WPI and curcumin (*K*_a_ and *n*) was investigated to analyze the fluorescence intensity behavior of CP‐treated WPI.

## MATERIALS AND METHODS

### Experimental design

The WPI samples were prepared by dispersing 10 g of powder onto polystyrene Petri dishes (90 mm in diameter), forming a uniform layer with an approximate thickness of 3 mm. The dishes were positioned between the electrodes of a plasma system, with a fixed gap of 15 mm. Plasma treatment was carried out using a dielectric barrier discharge system operating under atmospheric conditions in a parallel‐plate configuration. The equipment used was a PLS 0130 model (Inergiae, Brazil), consisting of two circular aluminium electrodes (90 mm in diameter) positioned in parallel. The samples were subjected to different excitation conditions, varying frequency (50, 500 and 950 Hz), applied voltage (10, 15 and 20 kV) and exposure time (5, 15 and 20 min), as detailed in Table 1. During treatment, environmental conditions were kept constant, and no modified atmosphere was applied, characterizing the use of nonthermal CP. After processing, the samples were packed in laminated polypropylene bags, sealed and stored at room temperature, protected from light and moisture until subsequent analyses.

**Table 1:** Experimental design to evaluate the effect of excitation frequency, voltage and time on WPI properties

Run	Excitation frequency (Hz)	Voltage (kV)	Time (min)
1	50	10	5
2	500	20	5
3	950	15	5
4	50	20	10
5	500	15	10
6	950	10	10
7	50	15	15
8	500	10	15
9	950	20	15

### Secondary structure

Fourier transform infrared spectroscopy (Cary 630 FTIR spectrophotometer, Agilent Technologies, USA) was used to analyze the changes in the secondary structure of native WPI and CP‐treated samples (WPI‐treated). After applying 0.1 g of powder to the crystal, 128 scans were conducted to examine the impact of CP on the samples using transmittance at wavenumbers between 4000 and 500 cm^−1^, with a resolution of 4 cm^−1^. The region between 1700 and 1600 cm^−1^ (amine I) was utilized to determine the secondary structure of proteins. The curves were smoothed (20 points) and subjected to curve deconvolution using the second‐derivative method (Savitzky–Golay using Origin 2022 PRO software, peak deconvolution tool) to calculate the areas of positive peaks. The *α*helix, *β*‐sheet, *β*‐turn and random coil structures were determined by the percentage of each representative peak.^12^

### Zeta potential

The surface charge (zeta potential) of WPI and WPI‐treated was determined using a Litesizer 500 (Anton Paar, Austria). For this, 1% (w/v) protein solutions were prepared with distilled water.

### Surface hydrophobicity

Surface hydrophobicity of the different samples was determined fluorometrically using an ANS probe.^13^ Samples were prepared by diluting to concentrations ranging from 0.005% to 0.050% (w/v) protein, using a molar ratio of 0.017:0.165 of citric acid–sodium phosphate buffer (pH 7). ANS probe solution (20 μL, 12.6 μg mL^−1^) was added to 200 μL of the diluted samples in a black 96‐well plate. The relative fluorescence index (*H*_0_) was measured at excitation and emission wavelengths of 371 and 5467 nm,^14^ respectively, after 15 min of being held in the dark at room temperature using a microplate reader (Thermoscientific/Multiskan SkyHigh).

### Carbonyl groups

The content of protein carbonyls was determined following the method of Levine *et al*.,^15^ with minor modifications. In brief, 0.4 mL of 2,4‐dinitrophenylhydrazine (DNPH; 10 mmol L^−1^ in 2 mol L^−1^ HCl) was combined with 0.3 mL of sample solution (20 mg protein mL^−1^, in ultrapure water) and incubated at room temperature for 60 min. An amount of 0.7 mL of trichloroacetic acid was added to the mixture and put in an ice‐bath for 10 min. After centrifugation at 10 000 × *g* for 3 min, the pellet was washed three times with 1 mL of ethyl acetate in ethanol (1:1 v/v) to remove DNPH excess. The washed solid was then dissolved in 0.5 mL of 6 mol L^−1^ guanidine hydrochloride, and the absorbance at 370 nm was recorded using a spectrophotometer. Results were expressed as a percentage relative to the carbonyl contents of the untreated control.^16^

### Free sulfhydryl

The free sulfhydryl group concentration in WPI was determined using the Ellman method with some modifications.^17^ In this study, 0.5 mL of WPI solution (1% w/v) was added to 2.5 mL of 8 mol L^−1^ urea in Tris–glycine buffer (10.4 g of Tris, 6.9 g of glycine, 1.2 g of EDTA per liter, pH 8.0). This was followed by the addition of 0.02 mL of Ellman's reagent which was prepared by dissolving 5′,5‐dithiobis(2‐nitrobenzoic acid) and DTNB in Tris–glycine buffer at a concentration of 4 mg mL^−1^. The solution was mixed well and incubated in the dark for 60 min. After that, its absorbance at 412 nm was measured using a UV–visible spectrophotometer. Concentration of free sulfhydryl groups (μmol SH g^−1^) was calculated from Eqn (1):

(1) $$\text{Free}\;\mathrm{SH}\;\left(\text{μmol}\;\mathrm{SH}\;g^{-1}\right)=\frac{73.52\;\times \;A_{412\mathrm{nm}}}{C}$$

where *A*_412nm_ is the absorbance at 412 nm, *C* is the protein concentration of WPI and the factor 73.53 is derived from 10^6^/(1.36 × 10^4^): 1.36 × 10^4^ is the molar absorptivity constant, while 10^6^ is for conversions from the molar basis to the mmol mL^−1^ basis and from milligrams of protein to grams of protein.

### Solubility

In summary, 3 g of each WPI‐treated was dissolved in 60 mL of distilled water. The Eppendorf tubes were shaken for 1 h at room temperature and centrifuged at 8000 × *g* for 10 min at 4 °C. The supernatant from each tube (2 μL) was pipetted into microplates, and protein quantification was performed using the Bradford assay. Protein solubility was calculated according to Eqn (2):

(2) $$\text{Solubility}\;\left(g\;{\left(100\;g\right)}^{-1}\right)=100\;\times \;\frac{{\left[\text{Protein}\right]}_{\text{final}}}{{\left[\text{Protein}\right]}_{\text{initial}}}$$

### WPI–curcumin interaction

The study of WPI–curcumin interaction was based on Khanji *et al*.^18^ with some modifications. Briefly, stock solutions of curcumin were prepared in ethanol (96%) at a final concentration of 1.0 mg mL^−1^ and stored protected from light at 4 °C. Phosphate‐buffered saline (PBS) at pH 7.4 was prepared with ultrapure water (1 L: 8 g of NaCl, 0.2 g of KCl, 1.44 g of Na_2_HPO_4_, 0.24 g of KH_2_PO_4_). WPI and WPI‐treated solutions (50 g L^−1^) were dispersed in PBS buffer and stirred overnight at 20 °C to achieve maximum protein hydration and stabilization. Curcumin was titrated into the WPI, yielding different solutions in a stepwise manner (0–17 μmol L^−1^), and fluorescence spectra were obtained using a spectrofluorometer (Nanolog, Horiba, Japan). A 280 nm excitation wavelength was used to collect the emission spectrum from 290 to 420 nm, with a 1 nm emission slit.

Fluorimetry data were fitted to a double‐logarithmic regression. This method is particularly useful in systems exhibiting static or dynamic quenching processes, as it allows for the calculation of the apparent binding constant (*K*_a_) and the number of equivalent binding sites (*n*) in the macromolecule. The model fitting, assuming a homogeneous interaction environment, is described by Eqn (3):

(3) $$\text{loglog}\;\left(\frac{F_{0}-F}{F}\right)=\log\left(K_{a}\right)+n\log\left(Q\right)$$

where *F* and *F*_0_ represent the fluorescence intensity of sample with and without curcumin, respectively; *Q* is the concentration of curcumin (mol L^−1^); *K*_a_ represents the binding constant of WPI with curcumin; and *n* represents the number of binding sites per WPI. The number of binding sites and binding constant were calculated from the slope and intercept of a double‐logarithm plot.

### Statistical analysis

Data analysis was performed using R software. Means were compared using analysis of variance (ANOVA) with the main effects of each variable (excitation frequency, voltage and time) at a significance level of *P* < 0.05.

## RESULTS AND DISCUSSION

### Secondary structure

The CP excitation frequency refers to the frequency at which free electrons in a plasma are excited, resulting in energy transitions. When observing the effect of frequency (Table 2), it was noted that at 500 and 950 Hz, the values of the different secondary structures of treated proteins differed from those of the control WPI. An increase in *α*‐helix and *β*‐sheet structures and a reduction in other structures, especially random coil, which was the most affected by CP treatment, were observed. An increase in the aggregated conformation of *β*‐sheet, along with a simultaneous decrease in random coil structure, may indicate protein aggregation after unfolding caused by CP exposure.^19^ CP does not induce covalent bond formation merely by molecular proximity; instead, it produces reactive radicals that subsequently undergo recombination. During this recombination stage, new covalent bonds are established, which may either involve existing hydroxyl groups or lead to the formation of new ones.

**Table 2:** Effect of CP process parameters on the secondary structure of WPI

Parameter		*α*‐Helix	*β*‐Sheet	*β*‐Turn	Random coil
Control		26.33 ± 0.27^b^	49.38 ± 0.57^b^	10.65 ± 0.54^b^	13.65 ± 1.02^a^
Frequency	50 Hz	26.42 ± 0.17^b^	49.66 ± 0.62^b^	11.48 ± 0.56^a^	12.44 ± 0.39^a^
	500 Hz	27.01 ± 0.24^ab^	52.08 ± 0.42^a^	10.37 ± 0.27^b^	10.54 ± 0.6^b^
	950 Hz	27.1 ± 0.18^a^	52.48 ± 0.13^a^	10.7 ± 0.23^b^	9.73 ± 0.33^b^
Voltage	10 kV	26.32 ± 0.32^a^	51.13 ± 0.42^b^	11.31 ± 0.11^a^	10.86 ± 0.6^a^
	15 kV	27.82 ± 0.05^a^	50.82 ± 0.86^b^	11.62 ± 0.45^a^	10.82 ± 0.72^a^
	20 kV	26.88 ± 0.33^a^	52.28 ± 0.3^a^	9.61 ± 0.11^b^	11.04 ± 0.47^a^
Time	5 min	26.68 ± 0.12^ab^	51.82 ± 0.44^a^	10.45 ± 0.33^b^	11.05 ± 0.29^ab^
	10 min	27.19 ± 0.19^a^	52.26 ± 0.28^a^	10.65 ± 0.3^b^	9.89 ± 0.69^b^
	15 min	26.65 ± 0.28^b^	50.14 ± 0.75^b^	11.44 ± 0.5^a^	11.77 ± 0.56^a^

Results are presented as mean ± standard deviation (*n* = 3). Different letters in the same column indicate significant differences (*P* < 0.05).

Voltage had a greater effect on modifying *β*‐structures, causing an increase in *β*‐sheet content and a reduction in *β*‐turn content as the applied plasma voltage increased (Table 2). Since changes in *β*‐structures can be indicative of protein aggregation,^20^ treatment at higher plasma voltages may favor protein–protein interactions and, consequently, their aggregation. Highly energetic free radicals likely promoted new covalent bonds within the protein structure due to the introduction of additional hydroxyl groups into the molecule. As a result, the protein's secondary structure becomes more organized. When this structure is more organized, it may hinder interactions with other molecules, as the main chemical groups available for binding may be hidden within the structure.

### Zeta potential

Table S1 (supporting information) presents the ANOVA test results for the main effects of the process parameters (excitation frequency, voltage and time) of CP on the following physicochemical parameters.

**Figure 1:**
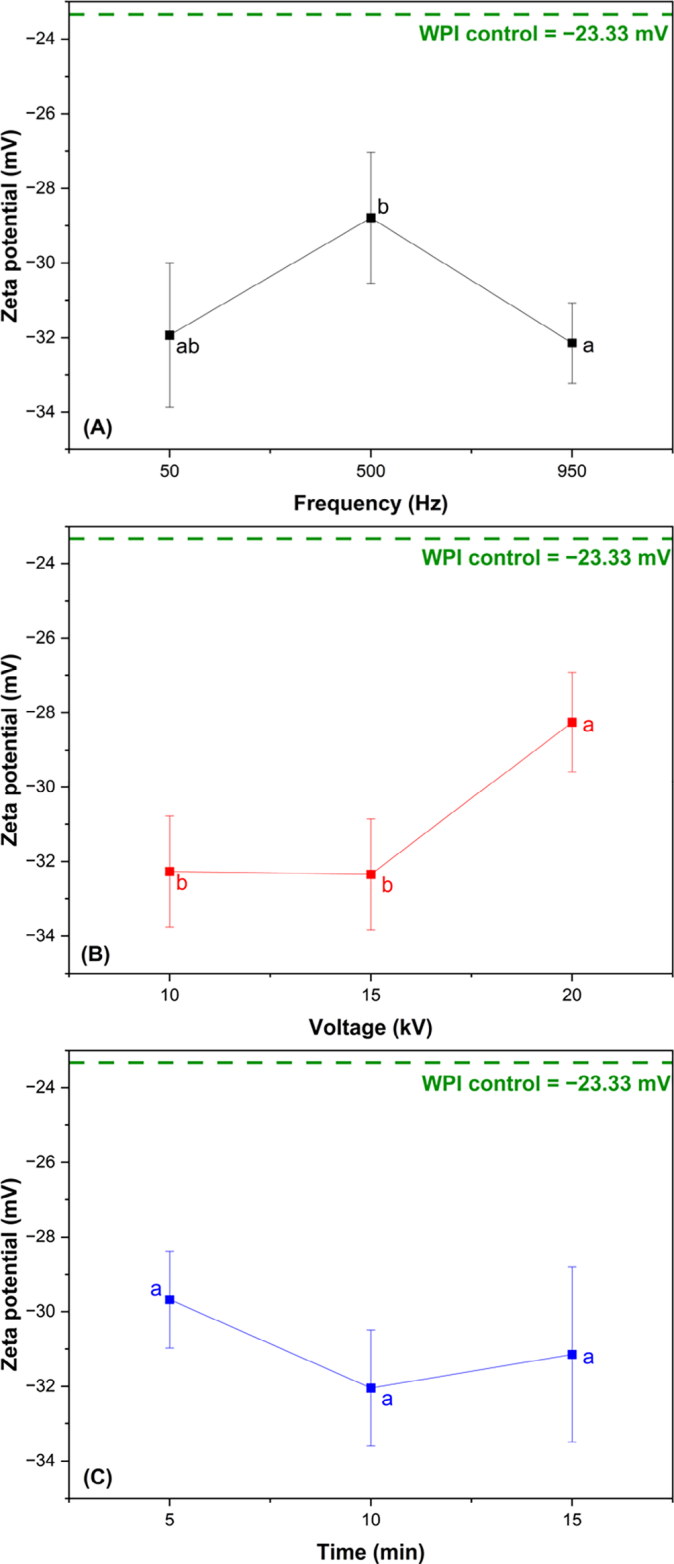
Effect of CP process variables on the zeta potential of WPI: frequency (A), voltage (B) and time (C). The results are presented as mean and standard deviation (*n* = 3). Different letters indicate significant differences (*P* < 0.05).

The effects of CP process variables on the zeta potential of WPI are presented in Fig. 1. Control WPI showed a zeta potential of −23.33 mV, and all CP treatments exhibited higher absolute values compared to the control. This result is noteworthy, as larger net charges can increase the repulsive forces between particles, reducing the tendency for aggregation and flocculation of these proteins.^8^

Regarding CP excitation frequency (Fig. 1(A)), 950 Hz showed higher absolute zeta potential values (*P* < 0.05) compared to other treatments and the control. Higher excitation frequencies had a positive effect on the formation of new carboxyl and carbonyl groups,^5^ potentially increasing the surface charge of CP‐treated biopolymers. As the CP excitation frequency increases, the power dissipated in food also increases, creating more radicals to cause oxidative damage to cells and organic molecules.^6^, ^21^

Higher excitation voltages significantly reduced (*P* < 0.05) the absolute zeta potential values (Fig. 1(B)). This may be related to the degree of protein denaturation. At lower excitation voltages (10 and 15 kV), the energy dissipated by the highly energetic CP radicals may have caused structural changes in WPI, exposing negatively charged chemical groups, thereby increasing the net surface charge of these proteins. These results are consistent with those of Liu *et al*.,^22^ who subjected ovalbumin fibrils to a voltage of 12 kV and found that this treatment increased the protein's positive charges through oxidation, thereby increasing the absolute zeta potential, even after protein heating. However, Wang *et al*.^23^ employed low‐voltage CP (50 W) on sunflower seed protein for treatment durations of 0, 1, 2, 3, 4 and 5 min, and observed that lower power led to a more negative potential, likely because CP only had enough energy to expose negatively charged amino acids and neutralize positively charged ones, resulting in a more negative zeta potential. At 20 kV, denaturation may have occurred to a greater extent, causing positive internal protein groups to interact with negative surface charges, reducing the absolute zeta potential value. Higher voltages induce higher concentrations of plasma‐reactive species and, consequently, higher reaction rates.^24^ Gong *et al*.^25^ also observed that more severe treatments (higher power and time) reduced the absolute zeta potential values of CP‐treated WPI.

The effect of time was not significant (*P* > 0.05) for modifications of the surface charge of WPI (Fig. 1(C)). Wang *et al*.^23^ studied plasma exposure time and reported that a 2 min exposure at 50 W significantly increased the zeta potential, while prolonged exposure reduced it. Both were directly related to the interaction of reactive species generated in plasma with the protein structure, exposing polar groups or degrading the protein molecule. However, in the present study, the energy transfer to the sample was higher, which may have damaged the protein structure and mitigated potential changes.

### Surface hydrophobicity

**Figure 2:**
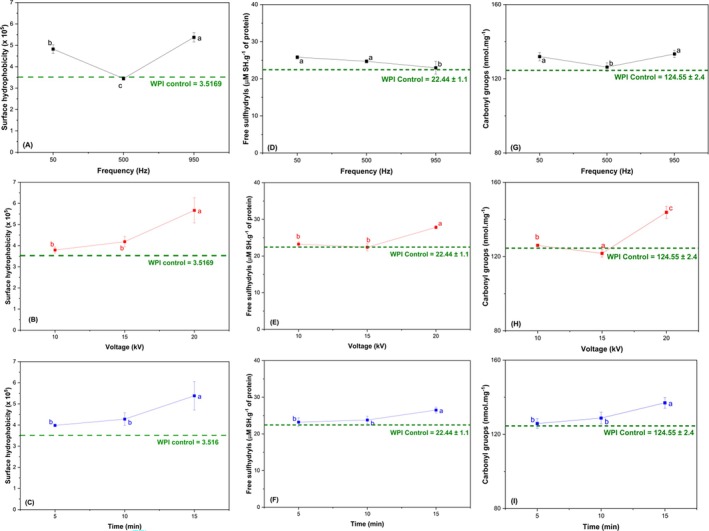
Effect of CP process variables on the surface hydrophobicity (A–C), free sulfhydryl groups (D–F) and carbonyl groups (G–I) of WPI: frequency (A, D, G), voltage (B, E, H) and time (C, F, I). The results are presented as mean ± standard deviation (*n* = 3). Different letters indicate significant differences (*P* < 0.05).

Figure 2(A)–(C) shows the results for the surface hydrophobicity of WPI treated with CP. In general, CP increased the hydrophobicity of the proteins, with only the 500 Hz frequency showing effects close to those of the control (Fig. 2(A)). The distribution of hydrophobic groups on the surface of proteins can be reflected by surface hydrophobicity. Additionally, exposure to or incorporation of hydrophobic groups into proteins can be caused by structural changes in the protein, thereby affecting the surface hydrophobicity.^26^ Zhang *et al*.^27^ found similar results, where CP promoted structural changes in a protein (as observed in Section Secondary structure), exposing intramolecular hydrophobic groups and increasing its surface hydrophobicity with increasing applied conditions.

The protein surface hydrophobicity increased with higher plasma voltage (Fig. 2(B)) and longer processing times (Fig. 2(C)). These effects can be explained by the interaction of ionized gases with the polymeric structure of proteins. When studying the effect of ozone (one of the gases produced during CP processing) on WPI modification, Segat *et al*.^28^ observed that longer exposure to ozone gas increased the hydrophobicity of proteins. The observed increase in surface hydrophobicity can be attributed to the unfolding of the molecular structure and a higher number of exposed hydrophobic groups after bombardment with high‐energy particles in prolonged treatments.^29^ CP can expose hydrophobic groups located inside the polymer chain, such as tryptophan and proline, transferring energy to the protein's surface region, causing the protein structure to depolymerize and stretch.^30^

### Free sulfhydryls

The results for free thiol groups are shown in Fig. 2(D)–(F). All process variables (frequency, voltage and time) were significant (*P* < 0.05) for the free thiol groups of WPI modified by CP. Increasing the frequency caused less formation of thiol group clusters in the polymeric structure of WPI, suggesting that CP can alter the structure of proteins (Fig. 2(D)). It is possible that specific excitation frequencies can generate reactive species with different energies sufficient to break or form disulfide bonds in the protein structure. In our case, increasing the frequency may have induced oxidation of cysteine residue pairs, resulting in new disulfide bonds. Ghorui^31^ observed that the higher the excitation rate, the better the ionization, and a larger group of ions and electrons participates in the response to the oscillating field, allowing greater energy dissipation. The increase in excitation frequency may have transferred enough energy for new disulfide bonds to be formed in the polymeric structure of the proteins through oxidative processes of cysteines, thereby stabilizing their tertiary structure (as evident from the results in Table 2).^32^ Liu and Xiong^33^ reported that cysteine residues are the first amino acids to be oxidized and can give rise to new chemical species or active binding sites. Additionally, a positive relationship between the formation of reactive oxygen species and excitation frequency was observed by Lin *et al*.^34^ Therefore, it is possible that at specific frequencies in our study, the production of reactive oxygen species was higher, resulting in the reduction of free thiol groups.

For plasma voltage (Fig. 2(E)) and processing time (Fig. 2(F)), the behavior was the opposite to that of frequency. The values of free thiol groups increased with higher voltage and processing time. Protein oxidation is always accompanied by a reduction in free thiol groups, where reversible forms (disulfide and sulfonic acid) or irreversible forms (sulfonic acid and sulfonate) can be generated under different conditions.^16^ With increased voltage, electrons gain more energy from the enhanced electric fields, and ionizing collisions between electrons and neutrals become more frequent.^35^ Tan *et al*.^36^ observed that sulfur‐containing amino acids and aromatic amino acids were sensitive to the reactive species produced by CP and are prone to oxidative modification, thus altering the thiol content.

Sharma and Singh^17^ reported a reduction in thiol content with increased exposure time, showing how reactive species, mainly from nitrogen, act on the breakdown of thiol groups present in whey protein. This result is contrary to what was found in this study, and this difference can be explained by the presence of frequency and voltage and their variations. The structural change caused by these two factors together may have led to the formation of new thiol bonds due to the oxidative process caused by CP reactive species. The reduction of –SH groups implies the formation of disulfide crosslinks (–S–S–), either intramolecular or intermolecular, and/or an irreversible effect leading to the formation of sulfinic and sulfonic acids.^16^, ^37^

The presence of free thiol groups can influence the surface hydrophobicity of a protein in various ways. For example, the formation of disulfide bonds can create crosslinks in the protein structure, affecting the exposure of hydrophobic residues on the surface. Moreover, the reactivity of thiol groups may be related to the presence of nearby hydrophobic residues, thus modulating the hydrophobic properties of the protein.

### Carbonyl groups

The results for the formation of carbonyl groups in WPI after the CP process are shown in Fig. 2(G)–(I). The CP process is highly oxidative due to the high energy of the reactive species formed during the excitation of ions and molecules present in atmospheric air. Several modifications in the protein structure result from the oxidative process, such as modifications in the side chains of amino acid residues, alterations in the polypeptide structure of the protein, oxidative cleavage of peptides, protein fragmentation, crosslinking, unfolding and conformational changes.^38^ Amino acids with NH or NH_2_ groups in their side chains are very sensitive to oxidation, and these groups are transformed into carbonyl groups during protein oxidation.^39^

### Solubility

**Figure 3:**
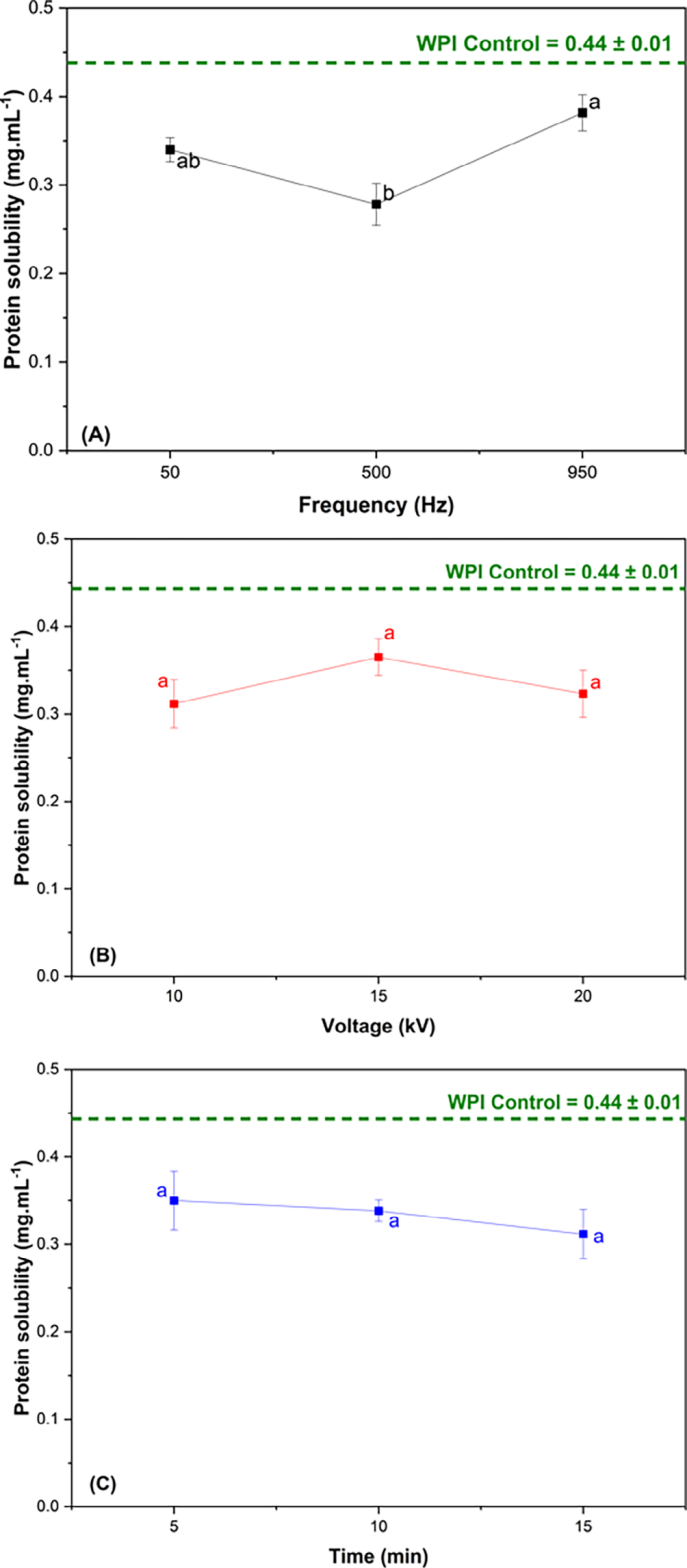
Effect of CP process variables on WPI solubility: frequency (A), voltage (B) and time (C). The results are presented as mean ± standard deviation (*n* = 3). Different letters in the same column indicate a significant difference (*P* < 0.05).

Solubility is one of the most relevant physicochemical properties of food proteins. As the primary solvent in most foods, water directly influences several techno‐functional characteristics such as solubility, foam formation, emulsification and gelation.^40^ Figure 3 shows the results of the main effects of CP process variables on the solubility of WPI. All treatments showed lower solubility values compared to the control. Ji *et al*.^29^ attributed the reduction in solubility of peanut proteins treated with plasma to the formation of supersaturated protein micelles and protein denaturation.

Excitation frequency significantly affected protein solubility (*P* < 0.05), with WPI exhibiting reduced solubility at 500 Hz. Tan *et al*.^36^ and Zhang *et al*.^41^ treated soybean protein and sheep whey protein, varying applied voltage and treatment duration, and reported enhanced solubility even under high‐voltage conditions. According to those authors, this effect was attributed to CP‐induced structural modifications, which increase the protein's exposed surface area and consequently improve its interaction with water. The present study showed that when placed as one of the variables, frequency directly affects the solubility result. Possibly, when the frequency is applied, the energy of ion excitation and reactive species was able to modify the secondary and tertiary structure, causing aggregations between proteins. Also, it is noted that the solubility results are a consequence of the various chemical and physical modifications observed in previous results. Changes in secondary structure, zeta potential, surface hydrophobicity and free thiol groups are related to the solubility results, highlighting trends in the effects of process variables.

### WPI–curcumin interaction

The intrinsic fluorescence properties of tryptophan, used as a measure of protein tertiary structure, are highly sensitive to the polarity of the microenvironment in which tryptophan is located.^42^ When the protein is in its folded state, tryptophan residues generally reside in the interior of the protein's core, a hydrophobic environment. In this context, they exhibit a high quantum yield and, consequently, an elevated fluorescence intensity.^42^

**Figure 4:**
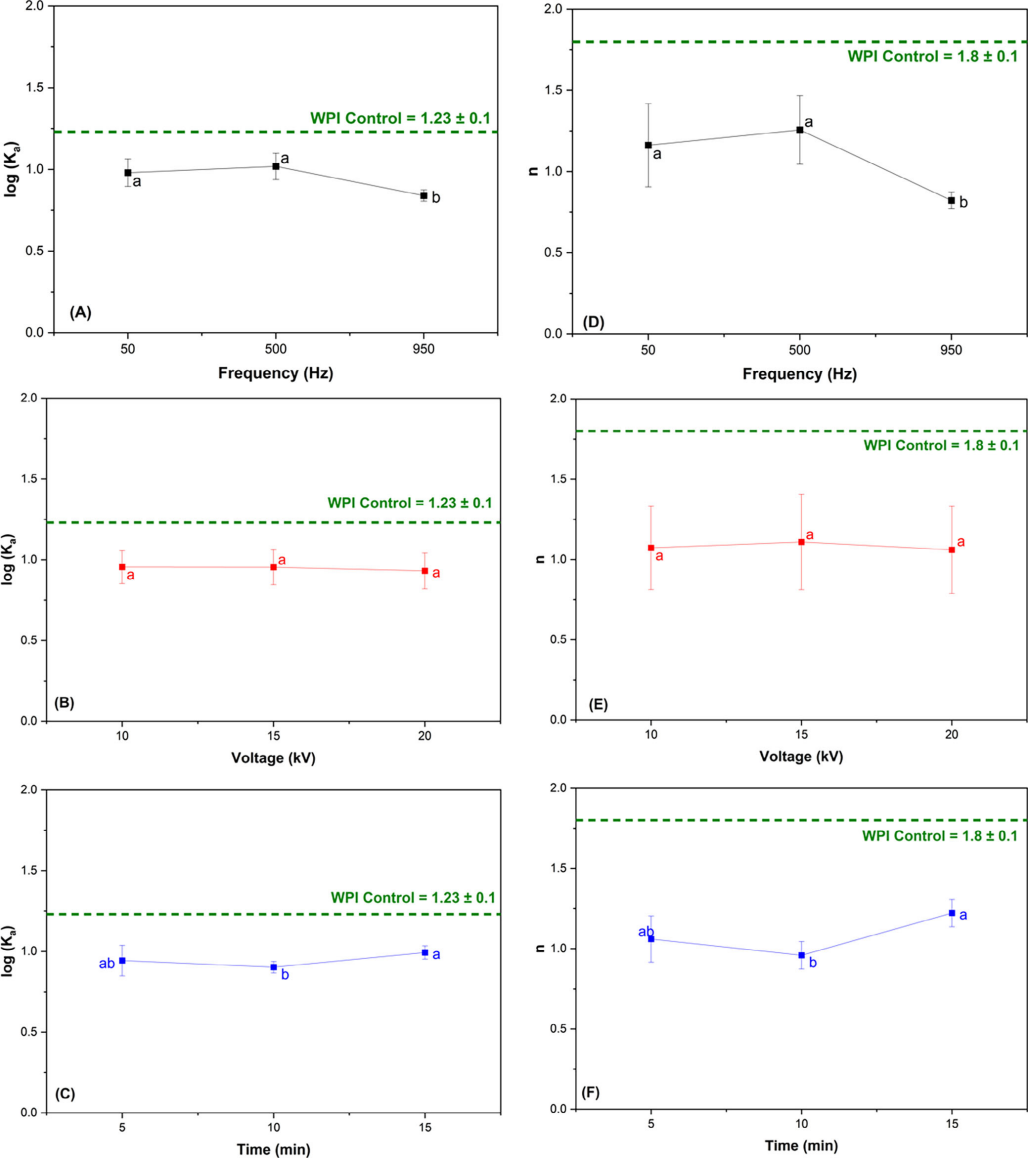
Parameters for interaction between CP‐modified WPI and curcumin: log(*K*_a_) (A–C) and *n* (D–F) for frequency (A, D), voltage (B, E) and time (C, F).

In our study, CP treatment led to two types of behavior: some treatments showed an increase in fluorescence intensity, while others showed a reduction (Fig. 4). That is, under specific plasma processing conditions, proteins can exhibit significant conformational changes that may influence their physicochemical and technological properties. At lower frequencies (50 and 500 Hz), there was a greater structural change in the proteins, causing fluorescent groups to be more exposed. In contrast, at higher frequencies (950 Hz), the fluorescence intensity values were lower compared to the control proteins.

The results found by Gong *et al*.^43^ demonstrate that a fluorophore used to bind with a suppressor also influences the fluorescence measurement. In this case, the aromatic ring of proanthocyanidin bound to tryptophan, leading to the extinction of fluorescence. Ji *et al*.^29^ observed that increased processing time with CP increased fluorescence intensity in pea proteins. They attributed this behavior to protein unfolding, which resulted in exposure of the chromophores after prolonged treatment.

Binding affinity is a measure of the interaction between a single biomolecule and its binding partner.^44^ All treatments showed lower *K*_a_ and *n* values compared to WPI control (Fig. 4). Proteins with more organized secondary structures (as observed from the results described in Section Secondary structure) may have fewer chemical groups available for binding with curcumin. This confirms the very similar *K*_a_ and *n* values for all samples compared to the control. Additionally, more hydrophobic proteins may aggregate due to an increased protein–protein interaction, reducing the active sites available for binding with curcumin.

### CP in innovation and further use for the dairy industry

Currently, one of the main research focuses is the development of alternatives to conventional milk preservation techniques, such as pasteurization and ultraheat treatment (UHT), which, although effective, induce alterations in the organoleptic properties of a product due to the denaturation and unfolding of casein micelles, impairing protein solubility and functional characteristics.^45^ The UHT process, in particular, subjects milk to more severe heat treatment, resulting in a lighter color, loss of vitamins and enzymes, hydrolysis of proteins and lipids, salt precipitation and a slightly sulfurous flavor caused by the release of SH groups from *β*‐lactoglobulin denaturation.^46^ Moreover, the high degree of protein denaturation in UHT milk prevents its use in cheese production, whereas CP treatment reduces solubility and surface hydrophobicity, promoting protein aggregation and gel formation, thereby expanding industrial applications and potentially enabling the unification of pasteurization and sterilization in a single process through a CP reactor.

## CONCLUSIONS

This study demonstrated the significant influence of CP treatment parameters excitation frequency, voltage and processing time on the structural and functional properties of WPI. The results revealed distinct effects on secondary structure, surface hydrophobicity, solubility and interactions with curcumin, emphasizing the tunability of CP treatment to achieve targeted protein modifications. This study underscores the potential of CP as a versatile tool for advancing protein functionality and aligns with the growing demand for sustainable and efficient food processing solutions. These findings pave the way for broader industrial applications of CP, including the development of innovative functional food ingredients and enhanced protein‐based products. Future research should focus on optimizing CP treatment conditions for different protein sources and exploring the synergistic effects of CP with other emerging technologies. Investigating the long‐term stability of CP‐treated proteins and their performance in complex food matrices will further elucidate the commercial viability of this technology.

## CONFLICT OF INTEREST

The authors declare that they have no known competing financial interests or personal relationships that could have appeared to influence the work reported in this paper. All authors have participated in (a) conception and design, or analysis and interpretation of the data; (b) drafting the article or revising it critically for important intellectual content; and (c) approval of the final version.

## Supporting information


**Data S1.** Supporting Information.

## Data Availability

The data that support the findings of this study are available from the corresponding author upon reasonable request.
