# Supplementary material for: Tailoring whey protein isolate properties through controlled cold plasma processing: excitation frequency, voltage and time as key variables
Source: J Sci Food Agric. 2025 Oct 11;106(2):1220–8. doi: 10.1002/jsfa.70245 (PMC12701304; doi:10.1002/jsfa.70245)
Supplement: Supplementary file 1 — Data S1. Supporting Information. [file JSFA-106-1220-s001.docx]

**Cold plasma and whey protein isolate: effect of excitation frequency, voltage and processing time on surface proteins properties performance**

Gabriel Oliveira Horta^1^; Paula Zambe Azevedo^1^; Breno Rodrigues de Souza^1^; Sueli Rodrigues^2^; Fabiano André Narciso Fernandes^3^; Daiana Wischral^1^; Paulo Cesar Stringheta^1^; Evandro Martins^1^; Pedro Henrique Campelo^1^

^1^ Department of Food Technology, Federal University of Viçosa, 36570-900, Viçosa, Brazil

^2^ Department of Food Engineering, Federal University of Ceará,

^3^ Department of Chemical Engineering, Federal University of Ceará,

**SUPPLEMENTAR MATERIAL**

**Table S1 - Univariate Tests of Significance for surface properties of WPI-modified by cold plasma: effect of excitation frequency, voltage and time**

| **Parameter** |  | ***SS*** | ***DF*** | ***MS*** | ***F*** | ***p-value*** |
| --- | --- | --- | --- | --- | --- | --- |
| Zeta Potential | **Intercept** | 25872 | 1 | 25872.6533 | 5379.92422 | 0 |
|  | **Excitation frequency** | 63.57 | 2 | 31.78 | 6.60 | 0.006 |
|  | **Voltage** | 98.44 | 2 | 49.22 | 10.23 | 0.0008 |
|  | **Time** | 25.68 | 2 | 12.84 | 2.67 | 0.0937 |
|  | **Error** | 96.18 | 20 | 4.80 |  |  |
| Surface hydrophobicity | **Intercept** | 5.58 × 10^12^ | 1 | 5.58× 10^12^ | 2810.05 | 0 |
|  | **Excitation frequency** | 1.77× 10^11^ | 2 | 8.86× 10^10^ | 44.5 | 0.0000 |
|  | **Voltage** | 1.75× 10^11^ | 2 | 8.79× 10^10^ | 44.22 | 0.0000 |
|  | **Time** | 9.78× 10^10^ | 2 | 4.89× 10^10^ | 24.59 | 0.0000 |
|  | **Error** | 3.97× 10^10^ | 20 | 1.98× 109 |  |  |
| Free sulfhydryl | **Intercept** | 16210 | 1 | 16210 | 11774 | 0 |
|  | **Excitation frequency** | 37.24 | 2 | 18.62 | 13.52 | 0.0001 |
|  | **Voltage** | 153.93 | 2 | 76.96 | 55.90 | 0.0000 |
|  | **Time** | 55.41 | 2 | 27.70 | 20.12 | 0.0000 |
|  | **Error** | 27.53 | 20 | 1.37 |  |  |
| Carbonyl groups | **Intercept** | 460136 | 1 | 460136 | 42507 | 0 |
|  | **Excitation frequency** | 253.57 | 2 | 126.78 | 11.71 | 0.0004 |
|  | **Voltage** | 2478.99 | 2 | 1239.49 | 114.50 | 0.0000 |
|  | **Time** | 601.45 | 2 | 300.72 | 27.780 | 0.0000 |
|  | **Error** | 216.49 | 20 | 10.82 |  |  |
| Solubility | **Intercept** | 2 | 1 | 2 | 1062 | 0.0000 |
|  | **Excitation frequency** | 0.032 | 2 | 0.016 | 8.61 | 0.0056 |
|  | **Voltage** | 0.009 | 2 | 0.004 | 2.50 | 0.1267 |
|  | **Time** | 0.004 | 2 | 0.002 | 1.231 | 0.3292 |
|  | **Error** | 0.020 | 11 | 0.002 |  |  |
| n | **Intercept** | 31.51 | 1 | 31.51 | 1099 | 0 |
|  | **Excitation frequency** | 0.93 | 2 | 0.46 | 16.39 | 0.0000 |
|  | **Voltage** | 0.01 | 2 | 0.01 | 0.20 | 0.8177 |
|  | **Time** | 0.31 | 2 | 0.16 | 5.54 | 0.0121 |
|  | **Error** | 0.57 | 20 | 0.03 |  |  |
| Ka | **Intercept** | 24.17 | 1 | 24.18 | 6272 | 0 |
|  | **Excitation frequency** | 0.16 | 2 | 0.08 | 20.66 | 0.0000 |
|  | **Voltage** | 0.01 | 2 | 0.01 | 0.40 | 0.6726 |
|  | **Time** | 0.08 | 2 | 0.02 | 4.86 | 0.0190 |
|  | **Error** | 0.08 | 20 | 0.01 |  |  |
